# Molecular Tools and Their Applications in Developing Salt-Tolerant Soybean (*Glycine max* L.) Cultivars

**DOI:** 10.3390/bioengineering9100495

**Published:** 2022-09-22

**Authors:** Adnan Rasheed, Ali Raza, Hongdong Jie, Athar Mahmood, Yushen Ma, Long Zhao, Hucheng Xing, Linlin Li, Muhammad Umair Hassan, Sameer H. Qari, Yucheng Jie

**Affiliations:** 1 College of Agronomy, Hunan Agricultural University, Changsha 410128, China; 2 Center of Legume Crop Genetics and Systems Biology/College of Agriculture, Oil Crops Research Institute, Fujian Agriculture and Forestry University (FAFU), Fuzhou 350002, China; 3 Department of Agronomy, University of Agriculture Faisalabad, Faisalabad 38040, Pakistan; 4 Research Center on Ecological Sciences, Jiangxi Agricultural University, Nanchang 330045, China; 5 Department of Biology, Al-Jumum University College, Umm Al-Qura University, Makkah 21955, Saudi Arabia

**Keywords:** abiotic stress, climate change, CRISPR/Cas9, genetic engineering, legumes, QTL mapping, omics

## Abstract

Abiotic stresses are one of the significant threats to soybean (*Glycine max* L.) growth and yields worldwide. Soybean has a crucial role in the global food supply chain and food security and contributes the main protein share compared to other crops. Hence, there is a vast scientific saddle on soybean researchers to develop tolerant genotypes to meet the growing need of food for the huge population. A large portion of cultivated land is damaged by salinity stress, and the situation worsens yearly. In past years, many attempts have increased soybean resilience to salinity stress. Different molecular techniques such as quantitative trait loci mapping (QTL), genetic engineering, transcriptome, transcription factor analysis (TFs), CRISPR/Cas9, as well as other conventional methods are used for the breeding of salt-tolerant cultivars of soybean to safeguard its yield under changing environments. These powerful genetic tools ensure sustainable soybean yields, preserving genetic variability for future use. Only a few reports about a detailed overview of soybean salinity tolerance have been published. Therefore, this review focuses on a detailed overview of several molecular techniques for soybean salinity tolerance and draws a future research direction. Thus, the updated review will provide complete guidelines for researchers working on the genetic mechanism of salinity tolerance in soybean.

## 1. Introduction

The world population is continuously rising, causing tremendous pressure on the global food supply [1]. Soybean (*Glycine max* L.) is one of the world’s most significant and fourth-largest legume crop [2,3]. Soybean is an important legume crop which provides important nutritional components such as milk, protein, and oil, and it has been widely used for industrial purposes [1,4]. With the passage of time, the global demand for soybean is continuously rising. Abiotic stresses affect crop growth and production worldwide. Soybean growth and yield are primarily affected by a series of environmental stresses [5]. Salinity stress is a disturbing environmental factor that severely affects soybean growth, yield, as well as quality. It affects plants during all growth stages [6,7,8,9]. About 434 million ha of land is facing the issue of salinity stress. Data showed that about 19.5% of cultivated land is affected with salt stress. Soybean is usually viewed as a crop more sensitive to salinity stress than other crops [10,11]. Salt toxicity arises from the absorption and accumulation of salt ions Na^+^ [12] and Cl^−^ in large concentrations [13,14]. Papiernik et al. [15] revealed that salinity stress is responsible for a 40% decrease in the yield of soybean and can lead to complete plant failure [13,16]. 

Due to the increased significance of soybean yields and issues produced by salinity stress, the development of tolerant soybean genotypes is gaining attention in the leading soybean breeding programs worldwide [17]. Legume’s growth and production are also influenced by salinity stress. Consequently, better genotypes with a high tolerance to salinity stress are compulsory to grow under salt-stressed conditions [18]. The development of salt-tolerant soybean depends on identifying the genomic regions that control the salinity tolerance [17]. Plants cope with salinity stress through complex molecular mechanisms, including genes and their pathways. Improving salinity tolerance in soybean requires a large gene pool to screen for tolerant genes [19]. Although a lot of research has been completed on the development of salt-tolerant cultivars of soybean, the genetic mechanism of this trait still needs to be fully uncovered to identify more genes and loci to be used in the breeding program [20]. Salinity stress tolerance is a polygenic trait, and improvement of this trait by conventional breeding methods is time-consuming and costly [21]. Quantitative trait loci mapping (QTL) is one of the most powerful techniques for identifying genomic regions that control tolerance to abiotic stresses. Numerous novel regions have been cloned and transformed to breed salt stress tolerance in soybean. Some untapped genes must be identified to use in QTL pyramiding programs [22,23]. Besides this, genetic engineering has a key role in developing transgenic soybean cultivars tolerant to salinity stress conditions [24]. Numerous studies have described the development of transgenic cultivars using genetic engineering technique [24,25]. 

Newly emerging techniques such as CRISPR/Cas9 have been used to develop resistance to several abiotic stresses in soybean, which shows their promising use for salinity stress tolerance [26,27]. The proteomics technique is employed to identify the proteins expressed during salt stress and that contribute to salinity tolerance in soybean [28]. Transcriptome analysis is a well-known technique which has a potential role in improving salinity tolerance in soybean. Until now, several genes controlling salinity tolerance in soybean have been identified. Both of these techniques mentioned above are omics techniques [19]. Until now, no comprehensive review has been presented which shows a detailed overview of several approaches for improving salinity tolerance in soybean. Hence, in this review, we have provided a combined summary of different molecular techniques and their importance in increasing salinity tolerance in soybean. This is followed by a brief explanation of current information about genetic factors and the genetics of salinity tolerance in soybean. Finally, we plan the possibility of connecting the existing knowledge with future research studies. This review will help to promote understanding about the molecular mechanisms controlling salinity tolerance in soybean to find a novel future research direction.

## 2. Effects of Salinity Stress on Soybean

Salt accumulation is a global environmental issue that largely affects crop production [3,29,30]. The effects of salinity stress are related with the higher concentration of salt ions, Na^+^ and Cl^−^, which hinder photosynthesis [31]. The accumulation of Na^+^ seems to be more harmful to *Glycine soja* [32]; however, the accumulation of Cl^−^ is more damaging for *Glycine max* [33]. Salinity stress decreased the function of nitrate reductase (NR), glutamine synthetase/glutamate synthase (GS/(NADPH), and glutamate dehydrogenase (GDH) in soybean [29]. Salinity stress reduces the leaf length, plant height, and leaf fresh weight of soybean [34]. Salinity stress causes oxidative stress in soybean by producing reactive oxygen species (ROS) [35]. Salinity stress reduced the growth and biomass of soybean cultivars [36] (Table 1). Çirka et al. [37] exposed soybean genotypes to 10,000 ppm NaCl and concluded that salinity stress positively affected seed germination and seedling emergence. 

Likewise, the availability of many significant nutrients such as nitrogen (N), phosphorus (P), and potassium (K) was reduced when soybean plants were treated with 86.30 mM NaCl [38]. Leaf reflectance and chlorophyll contents were decreased under salinity stress at a concentration of 40 mM [39]. In the same way, salt stress disturbed the level of abscisic acid and hydrogen peroxide (H_2_O_2_) [40]. Gibberellin (GA) is an essential plant hormone that controls the different phases of development and is involved in plant growth and organ development. Gibberellin activity is suppressed by NaCl, leading to an imbalance in the GA and ABA ratio [41]. He et al. [42] detected a noteworthy reduction in the stomatal conductance of soybean plants [42]. Salinity stress induces severe changes in soybean yields and quality. Salinity stress at a concentration of 6000 mg/L gradually decreased soybean yields by affecting yield traits such as the number of pods per plant and the number of seeds per plant [43]. Salinity stress also induces biochemical changes in soybean. The phospholipids profile and protein content of soybean declined after treatment with salt stress [44]. The oil content of soybean decreased after treatment with salt stress [45]. Time is needed to evaluate the soybean genotypes against different levels of salinity stress to understand the effects on various traits. 

**Table 1:** Salinity stress affects soybean’s morphological, physiological, and biochemical traits.

Effects	References
Salinity stress affected the activities of antioxidants	[46]
Caused ionic imbalance, and enhanced electrolyte leakage	[3]
Reduced growth and biomass in soybean cultivars	[36]
Salinity stress affected the seed germination percentage and seedlings growth	[37]
Reduced the NPK contents in plants	[38]
Total phenol contents reduced	[47]
Salinity stress decreased the contents of protein and phospholipids	[44]
Salinity stress decreased yield by affecting number of seeds/plant	[43]
Salt stress decreased the level of abscisic acid and hydrogen peroxide	[40]
Reduced oil contents	[45]
Reduced stomal conductance	[42]
Suppressed GA and ABA levels in cell	[41]
Generation of ROS	[35]
Leaf reflectance and chlorophyll contents were decreased	[39]
Reduced leaf length, fresh weight, and plant height	[34]

## 3. Genetic Mechanism of Salinity Tolerance in Soybean

Soybean, a glycophyte that is salt-sensitive, is greatly influenced by salinity at all growth phases. Soybean has long been studied to identify the molecular factors underlying salt tolerance because it is a salt-sensitive glycophyte [48]. It is usually a salt-sensitive species and needs genetic improvement to flourish in salt-affected soils [49]. The genetic mechanism of soybean is complex [50]. Several conventional breeding methods have been used to assess soybean’s tolerance to salt stress. Gene symbols such as *Ncl* and *ncl* were selected as indicators of being dominant and recessive for tolerant as well as susceptible cultivars [17].The genetic background of salinity tolerance was investigated by evaluating different crosses, which were significantly different in their level of salt tolerance. Studies showed that the F_1_ and F_2_ populations were marked as salt-tolerant when their parents were salt-tolerant. The soybean salt-tolerant genotype was crossed with a sensitive genotype, and the resulting F_1_ plants were salt-tolerant. The legacy of wild soybean genotypes was examined by evaluating the P1483463 accession. The inheritance of salt tolerance and relative gene alleles was studied using the crosses developed by crossing P1483463 and Hutcheson. The F_2_ offsprings of PI483463 × Hutcheson were segregated into tolerant and sensitive ratios of 3:1, whereas F_2:3_ lines were separated into a tolerant-to-sensitive proportion of 1:2:1.

F_2_ plants from the cross of PI483463 × S-100 separated into a tolerant-to-sensitive ratio of 15:1, demonstrating diverse genes from the two parents. Their outcomes exhibited that *Glycine soja* line PI483463 showed one dominant gene for salinity tolerance dissimilar from the gene of *Glycine max* line S-100 [51]. Likewise, a soybean F_2:3_ progeny was developed from a cross of (Tiefeng 8, a salt-tolerant genotype, and 85–140, a salt-sensitive genotype) to examine the genetics of salinity tolerance. The F_2:3_ progeny revealed a segregation ratio of 1:2:1, demonstrating the single gene action for salinity tolerance in Tiefeng 8 [52]. In the same way, Hua-tao et al. [53] used the recombinant inbred lines (RIL) population to study the pattern of the genetics of soybean salt tolerance. Salt tolerance is a polygenic trait in soybean controlled by numerous genomic regions, as proven by results of QTL and genome-wide association studies (GWAS). Further research is required to recognize the molecular mechanism of soybean’s tolerance to salt stress. Studying the nature of salt tolerance will help us choose a suitable breeding approach for developing salt-tolerant cultivars. 

## 4. Genetic Diversity 

**Figure 1:**
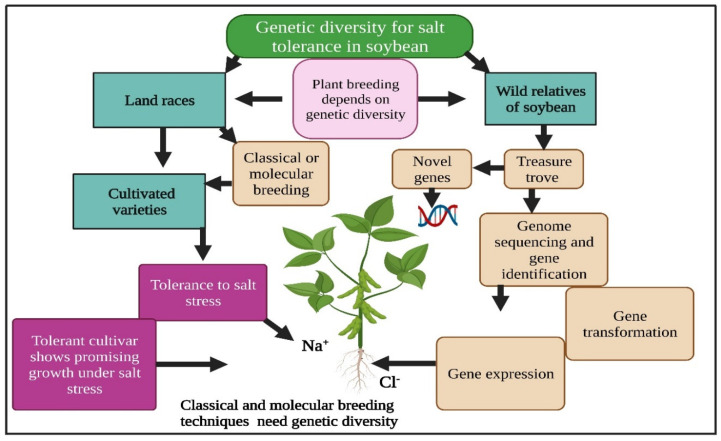
Genetic diversity has a key function in salinity tolerance in soybean. Soybean wild relatives are an important gene source for use in plant breeding programs. This Figure is created with BioRender.com.

Genetic diversity plays a key role in plant breeding programs. Plant breeding programs depend on the amount of genetic diversity. Various soybean germplasms have been screened, and a huge genetic diversity for salt tolerance has been recognized [17,54]. Genetic diversity is an essential condition for any breeding program (Figure 1). The screening of germplasm leads to identifying novel genes for salinity tolerance in soybean. Parker et al. [55] revealed that the contents of Cl^−^ in the leaves of sensitive genotypes were higher compared to tolerant cultivars, and susceptible cultivars had 37% less yield than tolerant cultivars. Yang and Blanchar [56] screened soybean genotypes and identified 19 Cl^−^-tolerant genotypes. Likewise, 2 more salt-tolerant lines have been recognized from a group of 257 soybean lines [57]. The wild soybean accessions ZH13 and BB52 were selected from different areas and placed under salt stress [58]. An additional wild soybean genotype, W05, was categorized as salt-tolerant [20]. Following this, 123 soybean accessions, including 6 wild accessions, were identified as salt-tolerant on the basis of data collected from the hydroponic experiment [59]. 

Xu D H et al. [60] assessed more than 600 soybean accessions in a greenhouse, comprising wild and cultivated soybean. Multiple genotypes showed high salt-tolerance values such as Chinese cultivar (Jindou 6) and Japanese wild soybean (JWS156-1). Recently, five soybean genotypes were grown in pots in a hydroponic culture. Genotypes were evaluated under a control as well as salt stress to assess the tolerance of genotypes to salt stress and their relatedness [54]. These genotypes were used to investigate the genetic diversity among simple sequence repeats (SSR). The maximum genetic distance (1.0) was detected among many genotype pairs with GC840 vs. a control. Five genotypes were arranged into two major groups based on cluster analysis. GC840 is recognized to be salt-tolerant, and Binasoybean-3 and Binasoybean-5 were clustered in the same subgroup [54]. These studies showed that genetic diversity is critical for identifying salt-tolerant cultivars in soybean [54]. Seeing the higher level of gene diversity in *Glycine soja* and its adaptations to severe environments, *Glycine soja* holds the excessive potential to advance its agriculturally important domesticated relative [20].

A list of salt-tolerant cultivars developed by conventional and molecular breeding is shown in Table 2. The higher genetic and phenotypic difference in soybean germplasms, comprising cultivated and wild species, proposes that the genetic development of salt tolerance is feasible. Further investigations are required to deeply understand the genetic diversity of salt tolerance among different soybean genotypes. High genetic diversity can be used to recognize the novel genes controlling salt tolerance in soybean. Wild parents should be conserved to protect genetic diversity for future use. Significant efforts should be made to slow down the degree of genetic erosion, which is the main factor responsible for the loss of diversity.

## 5. Mapping of QTL for Salinity Tolerance in Soybean

Breeding soybean to enhance salt tolerance is time-critical, as mentioned in a previous study [48]. QTL mapping for salt tolerance has been a major molecular technique to sustain soybean growth in salt-affected soils [48]. QTL mapping provides the opportunity to identify the targeted regions on chromosomes that govern salt stress tolerance in soybean [67,68,69]. Cheongja 3 and IT162669 cultivars were evaluated to detect the putative QTL for salt tolerance in soybean. Among the identified QTL, *qST6* was detected on chromosome 6, and *qST10* was detected on chromosome 10, which control the traits linked to ion toxicity under salinity stress. The region containing the QTL *qST6* harbors the earlier reported QTL flood tolerance *4-1*, where its syntenic blocks on chromosome 12 hold QTL related to iron deficiency chlorosis tolerance: *Fe effic 4-3*, *8-3*, and *11-3* (Table 3) [48]. In another study, an F_6_ recombinant inbred lines (RIL) population was used to detect the QTL for alkaline salt tolerance in soybean. An assessment of salt-tolerant QTL was performed using the salt tolerance ratio (STR) after treatment of the population with 180 mM NaHCO_3_ for three weeks. Significant QTL was detected on chromosome 17, which showed 50.2 and 13.0% of the total variances in both populations (F_6_ RIL and F_2_), respectively. These findings suggested that QTL for salt tolerance in the current study differs from the QTL for salt tolerance reported in previous studies [70]. A main salt-tolerant QTL surrounded by SSR markers was identified on chromosome 3 by multiple interval mapping analysis. The candidate gene for this QTL was *Glyma03g32900* and should be the gene for salt tolerance in Jidou 12 [71]. 

Do et al. [72] used a population of 132 F_2_ families and analyzed it for mapping salt-tolerant QTL. The estimation for salt tolerance was achieved by examining the leaf scorch score (LSS) and chlorophyll content ratio (CCR). In addition, a second region linked with salt tolerance for LSC was spotted and detected on chromosome 13 with a LOD score of 4.6. This QTL proposes the existence of a new gene governing salt tolerance and maybe stacked with an identified gene on chromosome 13 for enhancing salt tolerance in soybean [72]. An F_2:3_ mapping population was screened against salt stress tolerance under greenhouse conditions. A main QTL was detected on chromosome 3, and a minor QTL was detected on chromosome 19. The QTL on chromosome 3 has been verified and validated with previously reported QTL in soybean [73]. In another investigation, Ha et al. [74] evaluated the recombinant inbred lines (RIL) population to check its performance against salt stress and to identify the QTL controlling salt stress tolerance in soybean. SSR markers were used for molecular genotyping. Outcomes exhibited that salt-tolerant QTL was detected on chromosome 3. A subset of 66 iso-lines resulting from the same cross was evaluated to validate salt tolerance QTL recognized in the RIL mapping population. Results exhibited that this QTL was in the same chromosomal region as in the RIL population. This fine area can help in conducting further investigation for salt tolerance in soybean and the cloning of salt-tolerant genes [74]. 

To identify the salt-tolerant QTL, 96 recombinant inbred lines (RILs) population was evaluated under salt stress conditions. QTL analysis identified the major salt-tolerant QTL *qSTR3*, which exhibited 47.1% of the total variance. This QTL was validated using the NIL population, and the results confirmed that the lines having the FT-Abyara chromosome section at the QTL area were tolerant to salt stress. In contrast, lines without this segment were sensitive to salt stress [75]. Kan et al. [76] used 184 RIL and identified 11 QTL and 22 SSR for salt tolerance in soybean. Out of these 11 QTL, 2 loci, *qST-IR-8* and *qST-GR-8*, were consistent with previously reported QTL as indicated by the localization of SSR markers closely linked with QTL [76]. Do et al. [59] revealed that the salt-tolerant QTL *qNcl3* was mapped on chromosome 3. As per the reference soybean genome, there were seven projected genes in this 58.8 kb area of the main salt-tolerant QTL. The near-isogenic lines (NILs) harboring the tolerant allele of the QTL could raise soybean grain yield by 3.6–5.5 times in salt-affected fields [59]. Tuyen et al. [77] used the F_6_ RIL population to recognize the QTL *qSTR17* (Table 3) for salinity tolerance in soybean. The linkage mapping analysis discovered a major QTL with a large consequence for salt tolerance, and the maximum LOD score was noticed between the SSR markers GM17-12.2 and Satt447. Additionally, 10 static recombinant lines having chromosome segments of dissimilar lengths in the QTL area were selected from the RHL46 population. Two alkaline salt-tolerant NILs and their parents were used to validate the reported QTL, and results showed that major salt-tolerant QTL was located on chromosome 17 [77]. In another study, Zeng et al. [78] identified the salt-tolerant soybean lines using marker-assisted selection (MAS). An F_4:6_ mapping population was developed by crossing RA-452 and Osage. QTL analysis and composite interval mapping showed that a major chloride (Cl^−^)-tolerant QTL was validated and narrowed on chromosome 3 in NaCl and KCl treatments. A novel Cl^−^-tolerant QTL was detected on chromosome 15. This novel QTL was confirmed by comparison with earlier reported QTL and indicated the potential use of this QTL for accelerating the salt breeding program [78]. 

QTL analysis for salt tolerance in soybean provides little information about the number of QTL discovered. Extensive research studies using diverse mapping populations will help to identify the putative genomic regions controlling salt tolerance in soybean. The modified dose of salt stress should be used to screen the genotypes for their varying responses at different growth stages. This part of the review aims to combine the available published reports about QTL analysis for salt tolerance in soybean.

**Table 3:** Major salt-tolerant QTL identified in different soybean populations.

Population/Parents	QTL	Chromosome	References
Cheongja 3, IT162669	*qST6*	6	[48]
RIL	*qSTR3*	3	[71]
132 F_2_ families (Williams 82 × Fiskeby III)	*qLSC13*	13	[72]
F_2:3_ mapping population (Ozark × Jake)	*qLCC3*	3	[73]
RA-452 and Osage	*qLCC15*	15	[78]
RIL	*qNcl3*	3	[59]
RIL	*qST-GR-8*, *qST-IR-8*	8	[76]
F_4:6_ (RA-452 × Osage)	*qLCC13*	13	[79]
RIL	*qST3*	3	[74]
F_6_ RIL	*qSTR17*	17	[77]
96 RIL	*qSTR3*	3	[75]

## 6. GWAS for Salt Tolerance in Soybean

Numerous studies detected the novel QTL using the GWAS technique. Previously, a total of 283 soybean genotypes were screened against salt stress in greenhouse conditions. Leaf chloride concentrations and leaf chlorophyll contents were used as an indicator of salt tolerance. A low chloride content means genotypes are tolerant to salt stress and show a high photosynthesis rate. NaCl at the concentration of 120 mM was used to assess the performance of 283 diverse soybean plant introductions (PIs). Using GWAS, 45 single nucleotide polymorphisms (SNPs) representing nine regions on chromosomes 2, 3, 7, 8, 10, 13, 14, 16, and 20 were identified as being linked with leaf chloride concentrations. This study detected 31 SNPs positioned on chromosome 3 as potent salt-tolerant QTL. SNP on chromosome 2 was also in line with earlier reported SNPs for salt tolerance in soybean. The rest of the SNPs represent seven novel QTLs for salt tolerance in soybean. These identified SNPs were highly endorsed for marker-assisted selection (MAS) to breed salt-tolerant soybean cultivars [80]. Many studies showed novel genes for salt tolerance but did not fully explain the genetic variability. GAWS studies have been performed for salt stress tolerance in diverse soybean accessions using high-density SNPs. Likewise, another GWAS was completed in a subset of 234 accessions using 3.7 million SNPs. Three gene-based markers (GBM) of a known gene (*Glyma03g32900*) located on chromosome 3 were combined into two datasets. Leaf scorch scores (LSS) and leaf sodium content (LSC) were used to evaluate the salt tolerance among soybean lines. Several SNPs validated a major locus for salt tolerance located on chromosome 3 in both GWAS studies. Salt-20 and Salt11655 have the highest correlation with all studied traits [81]. GWAS is the most powerful way of recognizing the major genomic regions for salt tolerance in soybean. 

Cao et al. [82] performed GWAS to study the genetic variability for salt tolerance in soybean at the seedling stage. They used 281 diverse soybean accessions with 58,112 SNPs. In this study, two salinity tolerance indices, root fresh weight (ST-RFW) and root dry weight (ST-RDW), were used to assess the salinity tolerance in soybean. A total of 6 and 4 QTL were identified for ST-RFW and ST-RDW, respectively. The identified QTL, *qST.5.1* and *qST.16.1*, were associated with at least three stress tolerance indices on chromosomes 5 and 16. These QTL were described as major salinity-tolerant regions. All of these findings suggested that these potent regions could be used to improve salinity tolerance in soybean [82]. In another investigation, 192 diverse soybean lines were evaluated for salinity tolerance. Results showed that 62 SNPs signifying six genomic regions on chromosomes 2, 3, 5, 6, 8, and 18 were linked with salt tolerance in soybean. This study mapped 52 SNPs on chromosome 3 near the major QTL [22]. Zeng [79] conducted GWAS to identify the major regions for salt tolerance in soybean. Results showed the salt-tolerant QTL on chromosome 3 and revealed the eight novel QTL located on chromosome 2, 7, 8, 10, 13, 14, 16 and 20. Shi et al. [83] evaluated the 142 lines of cultivated soybean and 121 wild soybean accessions and identified genetic loci in wild soybean that are active during the seed germination stage under salt stress. They have identified 25 QTL by linkage mapping and GWAS analysis. These QTL significantly contributed to salt tolerance in soybean [83]. These QTL and linked SNPs would be useful for developing salt-tolerant soybean lines [83]. Zhang et al. [57] assessed the soybean population and studied an epistatic association mapping analysis for salt tolerance at the germination stage. A total of 83 salt-tolerant QTL were found at the germination stage. Out of 83 QTL, 19 were validated using an enriched compressed mixed linear model (E-cMLM) technique. However as per previous reports, there is limited information about QTL at the germination stage, which indicates that further studies are needed to identify more QTL for this trait [57].

Salinity stress has caused a significant reduction in soybean yields and quality worldwide. Identification of QTL provides a way of improving salt tolerance in soybean using MAS. There are certain unsolved issues (low allelic diversity and amount of recombination that occur during the creation of RIL place a limit) in developing salt-tolerant soybean genotypes. Using wild soybean accessions would be a fruitful option for the desired genomic region for salt tolerance. 

## 7. Identifying GWAS-Based Genes for Salt Tolerance in Soybean

Identifying GWAS-based genes controlling salt tolerance in soybean has been a prolonged approach. Previously, many GAWS were conducted to identify the major gene families and their role in salt tolerance in soybean; however, further studies will identify more genes and alleles for a detailed understanding of the genetic mechanism of this trait and strengthen our knowledge to improve breeding programs [84]. A GWAS was conducted to identify the major gene for soybean salt tolerance. A total of 117 GRAS genes on 20 chromosomes were identified and classified into 11 subfamilies. The results of RNA sequencing analysis revealed that most of the *GmGRASs* were expressed in 14 soybean tissues and involved in the response to abiotic stress [85]. In another study, 81 *DUF4228* genes were identified in soybean, which were named systematically based on their position on a chromosome. These genes were unevenly located on 20 soybean chromosomes. The expression profile of these genes was characterized by using RNA sequencing data under salt stress. These results showed that *DUF4228-70* plays an important role in the response to soybean salt stress [84]. The CrRLK1L proteins have a key role in salt tolerance in soybean, but their function is not yet fully understood. In a study, 38 CrRLK1L genes were identified in soybean. The relationship of these genes was further studied using phylogenetic analysis, which showed that these genes were arranged into three clusters: 1, 11, and 111. Chromosomal mapping indicated that these genes were located on 14 and 20 soybean chromosomes. Overexpression of these genes improved salt tolerance in soybean [86]. Shi et al. [83] conducted a GWAS analysis for salt tolerance in soybean. They evaluated the cultivated lines and wild soybean accessions under salt stress. The key genetic regions were identified based on QTL and SNPs. By gene-functional annotations of the W05 genome and salt-induced gene expression qRT-PCR analysis, *GsAKR1* was nominated as a candidate gene for salt tolerance at the germination stage in the wild soybean. These outcomes could aid in determining the genetic links of salt tolerance in wild soybean. They will support molecular marker-assisted selection (MAS) to breed salt-tolerant soybean cultivars [83]. Patil et al. [87] used whole-genome resequencing of 106 soybean lines and identified allelic variation in the promotor region of the *GmCHX1* gene. The detection of SNPs related to structural variants helped the design of six KASPar assays. These SNPs identified the salt-tolerant and salt-sensitive cultivars and increased the probability of developing salt-tolerant cultivars [87]. Recently, Dong et al. [88] used GWAS to identify the salt-tolerant loci (ortholog of *Arabidopsis GIGANTEA* (GI)) in soybean (Table 4). Loss of E2 functions reduced the flowering time and increased salt tolerance in soybean. The knockout mutant of E2 reduced the accumulation of ROS and improved the activity of antioxidant enzymes [88]. More GWAS can be useful to identify the new marker–traits association to develop a durable salt tolerance in soybean. The use of diverse soybean populations, different salt stress levels, and salt-tolerant cultivars would be critical for unfolding the genetic mechanism of salt tolerance in soybean. 

## 8. Genetic Engineering for Salinity Tolerance in Soybean

**Figure 2:**
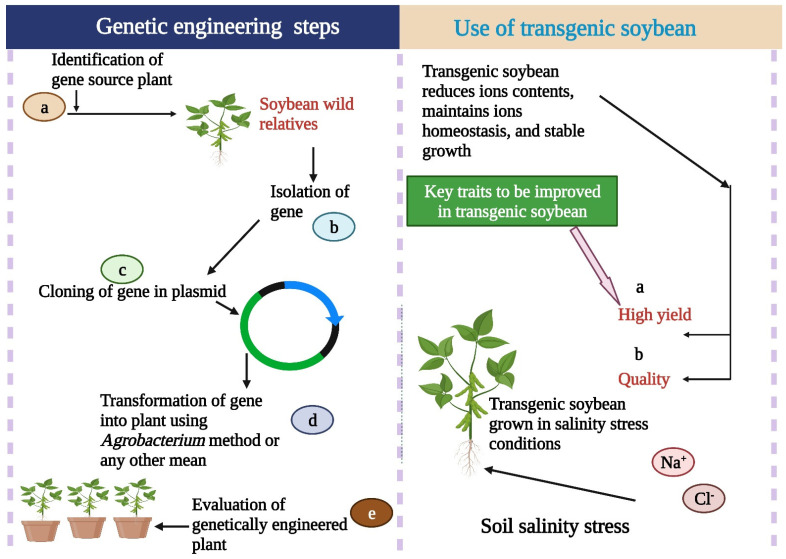
Genetic engineering has a major role in salinity tolerance in soybean. Transgenic soybean cultivars maintain growth and yield under salinity stress conditions. This Figure is created with BioRender.com.

Genetic engineering is a key technique in improving soybean salt tolerance (Figure 2) [89]. Identifying genes underlying tolerance mechanisms is essential for increasing salt tolerance in soybean. One of the extensively used approaches is genetic modification of cultivars for desired traits. Many research studies have proven the successful use of genetic engineering to develop salt-tolerant soybean. In an earlier study, *AgGlpF* was transferred into the cultivar Williams 82 genome to develop salt tolerance. The soybean cotyledonary node transformation technique was used to transfer the gene. PCR and RT-PCR results showed that *AgGlpF* was effectively combined into the soybean genome and showed significant expression. Later, transgenic lines were subjected to salt stress conditions, and results showed that transgenic lines exhibited a significant tolerance to salt stress compared to the wild type. These results showed the stable transformation and expression of *AgGlpF* (Table 5) in the soybean genome and its response to salt stress [90]. Ren et al. [91] studied the role of the soybean salt-tolerant gene *GmST1* (Table 5) and concluded that this gene reduced ROS production and increased sensitivity to ABA under salt stress situations [91]. This may be an ideal gene for the engineering of salt tolerance in soybean and *Arabidopsis*. Likewise, *GmPIP1;6* expressions were studied in the soybean’s roots and reproductive tissues. After treating soybean plants with 100 mM NaCl, the gene expression level increased in roots and leaves. *GmPIP1;6* gene expression was studied under salt stress and normal conditions. These studies showed the role of transgenic lines under salt stress conditions [92]. 

The salt tolerance of soybean cultivar NY-1001 was assessed by the transformation of gene *StP5CS* using the *Agrobacterium* transformation method. Soybean transgenic plants showed different ratios in the T_1_ generation. The T_2_ and T_3_ homozygous lines were examined for salt tolerance in a pot and hydroponic conditions. Overexpression of *StP5CS* confirmed salt tolerance in T_2_ and T_3_ homozygous lines. The gene *StP5CS* significantly increased the plant height, leaf area, chlorophyll content, and number of fresh pods of T_2_ and T_3_ compared to the control one [93]. Another salt-tolerant gene, *TaNHX2*, was transferred into soybean using *Agrobacterium rhizogenes* strain K599. Soybean plants were exposed to salt stress, and results showed that transgenic plants showed high salinity tolerance under the 200 mM NaCl. In contrast, plants under control conditions died within 15 days of treatments. Transgenic plants showed enhanced biomass, flowers per plant, and a long survival rate under salt-stressed conditions. These results indicated that *A. rhizogenes*-mediated transformation system could be used as an effective tool for the rapid transformation and investigation of a candidate gene in soybean [65].

Nie et al. [94] studied the function of the gene *GmsSOS1* for drought tolerance in soybean. This gene was isolated from soybean and enhanced salt tolerance in *Arabidopsis* [94]. Recently, the function of the gene, *GmNFYA* was studied in transgenic soybean under salt stress conditions. *Agrobacterium tumefaciens*-mediated cotyledon node transformation was used for gene transformation in soybean. Transgenic lines with an overexpression of *GmNFYA* showed salt tolerance due to the activation of stress-responsive genes [95]. In another study, the *PgTIP1* gene was transformed into soybean for salt tolerance. Results revealed that transgenic soybean lines exhibited high salt tolerance due to overexpression of the *PgTIP1* gene. The results exhibited that the salt-stressed *PgTIP1*-transgenic lines (L19 and L29) developed better leaf stomatal movement, a lower absorption rate, and the transport and accumulation of salt ions Na^+^, Cl^−^. Several stress-responsive genes were expressed as a result of the salt tolerance increase in soybean [64]. 

Cheng et al. [23] studied the role of soybean gene J in salt tolerance. *J* expression was induced by salt stress, and its protein was located in the nucleus. Overexpression of J enhanced salt tolerance in soybean (*NIL-J* mutant). *Agrobacterium rhizogenes* strain K599 was used to transfer the transgene [23]. The transgenic plant showed salt tolerance, as indicated by the genetic analysis of J [23]. In another study, a salt-tolerant locus, *GmSALT18*, was identified in soybean using two F_2:3_ mapping populations. Soybean wild line NY36-87 comprises salt-tolerance-related gene *GmSALT18*, provides genetic material and a new locus for breeding salt-tolerant soybean, and could be an ideal candidate for genetic engineering [96]. Using wild soybean relatives would be highly useful to transfer the salt-tolerant genes to accelerate the genetic engineering program. 

Wang et al. [97] increased the expression of a salt-tolerant gene, *GmBIN2*, in soybean under salt stress conditions. To examine the expression of *GmBIN2*, transgenic soybean hairy roots were generated. Results of a soybean hairy root assay revealed that overexpression of *GmBIN2* significantly increased the relative root growth compared to control plants (Table 5). *GmBIN2* also upregulated the stress-responsive genes in transgenic soybean hairy root [97]. To decrease the effects of salinity stress faced by soybean crop, Karthik et al. [98] transformed soybean cv. PUSA 9712 via straight organogenesis with the marker-free construct of *p68* gene using the *Agrobacterium*-mediated transformation method. Soybean transgenic plants showed a higher rate of photosynthesis, stomatal conductance, and CO_2_ assimilation than control plants. Soybean T_1_ plants showed higher K^+^ and lower Na^+^ contents. They also assessed the yield performance of transgenic soybean plants in a greenhouse under salt stress conditions. Present findings suggested that *p68* could be a candidate gene for developing salt-tolerant soybean cultivars [98] (Table 5). Overall, these findings suggested the potential role of genetic engineering in breeding salt-tolerant transgenic soybean cultivars [1,99]. Further studies on the soybean genome will help to address the challenges of genetic transformation in soybean. Salt stress is a polygenic trait, and unfolding of the genetic mechanism of this trait will be helpful in the identification of salt-tolerant genotypes of soybean.

**Table 5:** Genetic engineering for salt tolerance in soybean.

Gene	Role	Transformation Method	References
*GmLecRlk*	Enhanced fresh weight, proline content, as well as catalase activity	*Agrobacterium rhizogenes*, *EHA105*	[99]
*GmNFYA*	Induced expression of salt-responsive genes	*Agrobacterium tumefaciens*-mediated cotyledon node transformation	[95]
*AgGlpF*	Enhanced salt tolerance	Soybean cotyledonary node transformation method	[90]
*J* (ortholog of *AtELF3*)	Controlled the expression of stress-related genes (*GmWRKY27* and *GmNAC*)	*Agrobacterium rhizogenes* strain K599	[23]
*p68*	Increased photosynthetic rate, stomatal conductance, and CO_2_ assimilation	*Agrobacterium tumefaciens* strain EHA105	[98]
*PgTIP1*	Developed better leaf stomatal movement as well as water–gas exchange capabilities	Pollen-tube pathway method	[64]
*GmST1*	Reduced ROS production and increased sensitivity to ABA	*Agrobacterium tumefaciens* GV3101	[91]
*GmsSOS1*	Improved seed germination and seedling growth	*Agrobacterium tumefaciens*	[94]
*StP5CS*	Increased the plant height, leaf area, chlorophyll contents, and number of fresh pods	*Agrobacterium*-mediated cotyledonary-node method	[93]
*GmPIP1;6*	Enhanced leaf gas exchange rate	*Agrobacterium tumefaciens*	[92]
*TaNHX2*	Enhanced biomass, flowers per plant, and long survival rate under salt stress conditions	*Agrobacterium rhizogenes*-mediated transformation	[65]
*GmBIN2*	Increased the relative root growth and upregulated stress-responsive genes	*Agrobacterium rhizogenes* K599	[97]

## 9. CRISPR/Cas9-Mediated Salt Tolerance in Soybean

**Figure 3:**
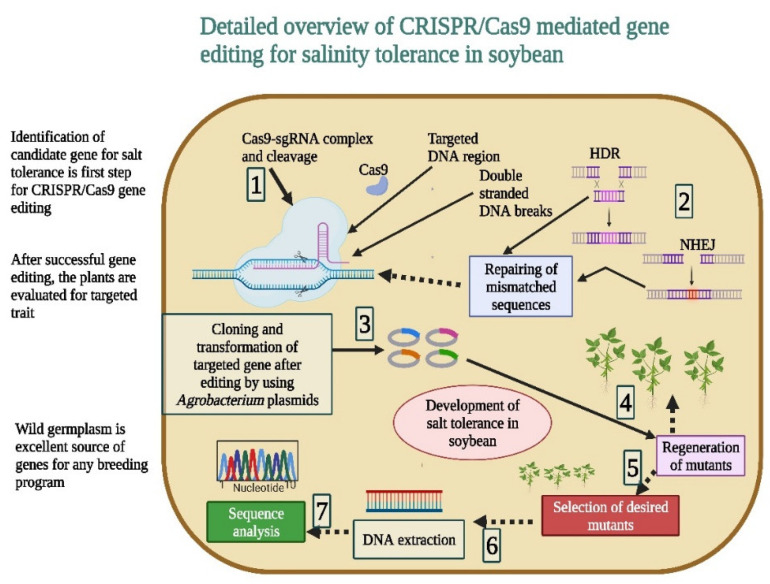
CRISPR/Cas9-mediated gene editing plays a key role in the development of salt-tolerant soybean genotypes. It develops the transgene-free cultivars to ensure biosafety regulations. The editing of genes for salt tolerance makes this tool highly efficient with less errors. This Figure is created with BioRender.com.

CRISPR/Cas9-mediated gene editing has become an important tool in modern-day plant breeding (Figure 3) [100,101] which is creating significant variations in genomes regardless of any biological barrier [102]. CRISPR/Cas9 has been used to knock out hundreds of genes and increases tolerance to abiotic stresses. For the success of CRISPR/Cas9 applications, single-guided RNA (sgRNA) must be expressed in the host organism to edit multiple genes [103,104]. Multiple genes can be edited if all genes have the same sequence. Salinity stress is caused by soil salinization, a core issue that decreases soybean yields and growth. Numerous genes have been recognized for salt tolerance in soybean and have been characterized. It is therefore important to apply newly emerged gene-editing tools such as CRISPR/Cas9 to edit the targeted gene in soybean. Du et al. [63] studied soybean plants with an overexpression of *GmMYB118*, which showed improved tolerance to salt stress. These plants were generated via the *Agrobacterium rhizogenes*-mediated transformation method. CRISPR/Cas9-based generated mutants (*GmMYB118* mutants) showed higher salt tolerance compared to the control. CRISPR/Ca9-based generated mutants showed higher proline and chlorophyll contents [63]. 

Recently, Sun et al. [62] employed CRISPR/Cas9 to alter the *GmNHX5* in soybean to improve salinity tolerance. CRISPR/Cas9-based generated mutants with an overexpression of *GmNHX5* showed an improved expression of *GmSOS1* and *GmSKOR*, a higher K^+^/Na^+^ ratio, and increased viability when exposed to salt stress. These results showed a potent candidate gene for salt-tolerant germplasm [62]. Likewise, in another study, the knockout of *GmAITR* genes by CRISPR/Cas9 increased salinity tolerance in soybean. Results of RT-PCR analysis exhibited that *GmAITRs* expression increased in response to salt treatment and ABA. The successful knockout of six *GmAITRs* resulted in the generation of Cas-free *gmaitr36* and *gmaitr23456* mutants in soybean. Seed germination and a seedling growth assay were completed, and it was found that *gmaitr* mutants exhibited tolerance to salt stress. A field experiment was conducted to assess the salinity tolerance of the mutants. These outcomes recommend that the mutation of *GmAITR* genes using CRISPR/Cas9 is an effective way to improve salinity stress tolerance in soybean [61]. Likewise, the CRISPR-Cas9 system was used along with the overexpression technique. It was found that *GmNAC06* increased the accumulation of proline to reduce the negative effects of ROS, and it could maintain the Na^+^/K^+^ ratios in hairy roots to retain the ions homeostasis [6]. 

CRISPR/Cas9 has been successfully used for precise genome alteration of soybean. Until now, no successful studies have been reported on the use of this tool in the mutation of wild soybean (*Glycine soja*), which is a soybean ancestor and rich source of stress-responsive genes. Niu et al. [105] successfully applied CRISPR/Cas9 to edit the genes *GsSOS1* and *GsNSCC* with 28.5 and 39.9% mutation frequencies, respectively. The mutation of both genes altered the transcription profile in mutant roots. Meanwhile, many different genes involved in various cellular functions were identified. These findings supported the theory of successful gene mining as well as functional analysis in wild soybean [105]. These results suggested the use of CRISPR/Cas9 in transgenic wild soybean under salinity stress [6]. Overall, the use of CRISPR/Cas9 in improving soybean’s salt tolerance is limited and needs more studies. We endorse using new editing systems such as base editing and prime editing, which are bringing about a revolution in the field of agriculture. CRISPR/Cas9 applications in wild soybean would improve soybean’s tolerance to numerous abiotic stresses [105]. These studies on CRISPR/Cas9-based gene editing in soybean opened the door to extend the use of this tool to develop tolerant cultivars against abiotic stresses. More studies are needed to employ the novel CRISPR/Cas9 gene manipulation systems to accelerate the speed of molecular breeding in soybean.

## 10. Evidence of Transcription Factors (TFs) Analysis for Salt Tolerance in Soybean

TFs are proteins which control gene expression under several stress conditions. There are several successful studies on the use of TFs for the improvement of salt tolerance in soybean. However, the complete genetic mechanism and regulatory pathways of TFs are still not fully discovered. WRKY is one of the most significant families of TFs and regulates plant growth and development under salt stress; however, information about this family is not broadly available. A salt-tolerant gene, *GmWRKY12*, which is about 714 base pairs (bp) in length, encoded 237 amino acids and classified into WRKY II. *GmWRKY12* was predominantly expressed in different tissues under control conditions and highly expressed in salt stress conditions. This protein was accountable for salinity tolerance in soybean [106]. The activation of TFs and DNA methylation are important plant strategies to counter salt stress. Zhang et al. [107] studied the salinity-induced expression of the TF encoding gene *GmMYB84*, which relies on DNA methylation. Plant with an overexpression of *GmMYB84* (Table 6) outperformed when exposed to salt stress. Plants showed a high germination rate, root elongation, membrane integrity, and a low K^+^ level [107]. In the same way, 188 genes of the WRKY family were identified in soybean. These WRKY genes were classified into three major groups (11, 1, 111). Results of RT-qPCR showed that in the whole soybean plant, 66 *GmWRKYs* revealed different expression patterns under salt stress [108]. 

A rice TF, *OsDREB2A*, which belongs to the subfamily DREBP, is responsible for salt tolerance in soybean, and its expression is induced by salt stress. Overexpression of *OsDREB2A* in soybean increased salt tolerance by increasing soluble sugar content and proline contents and enhancing the expression of some stress-responsive genes. Transgenic soybeans outperformed under salt stress compared to the wild types (WT) [109]. In another study, the NAC gene *GmNAC06* was cloned and characterized for salt tolerance in soybean. Expression analysis was completed, and the results showed that salt stress could affect the expression level of this gene. Results showed that *GmNAC06* caused proline and glycine betaine (GB) accumulation and reduced the ratio of Na^+^/K^+^ [6]. Another WRKY TF gene, *MsWRKY11*, was isolated from alfalfa. The soybean plants that overexpressed *MsWRKY11* showed salt tolerance at the seedling stage. The transgenic soybean plants showed a superior phenotype compared to wild-type. *MsWRKY11* enhanced chlorophyll contents, soluble sugar, and catalase activity. Besides these, transgenic plants had a higher number of pods per plant, seeds per plant, and 100-seed weight than WT [66]. The involvement of the *GmCYP81E1* gene of *GmMYB183* TF enhanced soybean salt tolerance by increasing the rate of flavonoid biosynthesis [110]. A novel bHLH *TF*, *GmbHLH3,* was recognized in soybean. Soybean plants treated with NaCl showed an overexpression of *GmbHLH3*. The overexpression of *GmbHLH*3 improved the accumulation rate of Cl^−^ and NO_3_^−^ in roots, hence preventing their transport to shoots by maintaining the lower ratio of Cl^−^ and NO_3_^−^ in plants [111].

The TGA TFs play an important role in salinity tolerance in soybean. In an earlier study, 27 TGA genes were identified in soybean. Among the 27 TGA genes, *GmTGA17* expression was induced by salt stress. This gene was verified by a promoter–GUS fusion assay. The *GmTGA17* gene encodes for nuclear-localized proteins. Expression analysis showed that *GmTGA17* enhanced salt tolerance in hairy soybean roots. Likewise, physiological traits analysis showed that chlorophyll and proline contents were enhanced under salt stress in soybean seedlings [112]. A member of the NAC TF family was characterized for salt tolerance in soybean. Results of qRT-PCR revealed that *GmNAC15* expression was induced under salt stress conditions in roots and leaves of soybean, and its overexpression increased salt tolerance. It also regulated the expression of several stress-responsive genes [113]. A member of the basic leucine zipper (bZIP), *GmFDL19*, increased tolerance to salt stress in soybean. Its expression was highly induced by salt stress. Transgenic plants exhibited a higher expression of *GmFDL19*, as indicated by their higher shoot weight, plant height, and germination rate. Transgenic plants also showed significantly lowered Na^+^ ions compared to the wild types [114]. TGA is a subfamily of bZIP TF and plays a key role in the salt stress response in soybean. Another TGA gene, *GmTGA13,* was cloned, and its expression and cellular localization were measured. Genes were transformed using the *Agrobacterium* transformation method; transgenic plants were treated with salt stress, and their physiological traits were measured. Overexpression of *GmTGA13* leads to the absorption of K^+^, regulation of ions’ homeostasis, and activation of several stress-responsive genes [115].

A class-b heat shock factor improved salt tolerance in soybean through flavonoid accumulation and by inhibiting the activity of the *GmNAC2* gene. These results discovered the mechanism of HSFB2b in soybean under salt stress. Its promoter variations were recognized, and the haplotype with high activity may be accepted for breeding better soybean cultivars modified for stress circumstances. The wild soybean types (Y20 and Y55) could be a source of novel tolerant genes for salt breeding programs [116]. All these findings showed that TF families and their members are a potential target of CRISPR/Cas9, genetic engineering, and other molecular breeding tools used to develop salt-tolerant soybean cultivars [116]. 

The soybean dehydration-responsive bindings proteins (BREBs), which belong to the AP2 family, are important TFs that mediate the salt stress response in soybean. The earlier *GmDREB6* gene was transformed into soybean using Agrobacterium plasmid. The overexpression of *GmDREB6* in soybean enhanced the transcriptional level of the *GmP5CS* gene and proline accumulation. In transgenic soybean, proline contents were increased under salt stress compared to control plants [117]. Zhang et al. [99] conducted a qRT-PCR analysis to study the expression of the *GmLecRlk* gene in soybean roots under salt stress. The gene was introduced into the soybean genome by the *Agrobacterium rhizogenes* transformation method. Soybean lines with an overexpression of *GmLecRlk* had higher fresh weight, proline content, and catalase activity. Results revealed the role of *GmLecRlk* in enhancing the scavenging capability of antioxidants in soybean plants [99]. The homeodomain leucine zipper (HD-Zip) transcription factor family plays a key role in salt tolerance in soybean. The HD-Zip gene family members were identified in the soybean cultivar (William 82), and their expression was studied under salt stress. These members expressed unique expression patterns across all stress conditions [118] (Table 6). Results showed that William 82 could be used as a source of the novel gene to develop salt-tolerant cultivars [118]. 

**Figure 4:**
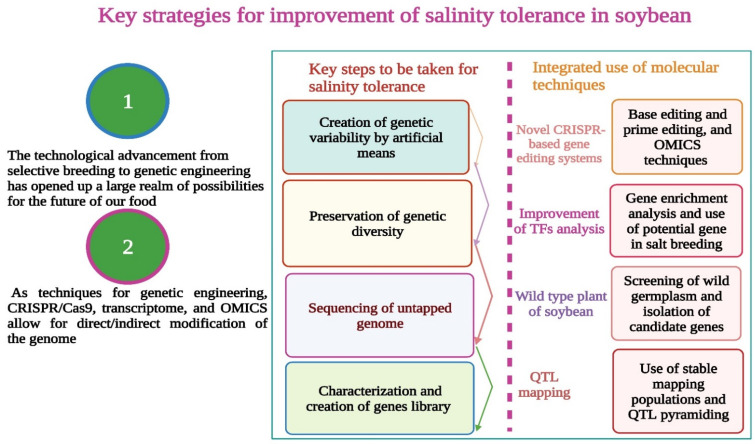
Salinity tolerance in soybean can be improved by using novel techniques such as, CRISPR/Cas9, genetic engineering, base editing, prime editing, genes from wild relatives of soybean, QTL pyramiding, and TF analysis. This Figure is created with BioRender.com.

The detailed analysis of TFs in soybean showed a promising way of improving salt tolerance by increasing the expression of TFs under salt stress conditions. Different TF families still need to be deeply explored to identify the TFs and their role in salt tolerance. All TF families have not yet been fully characterized and need further study to understand their regulatory network underlying soybean salt tolerance. This information sheds light on the improvement of salinity tolerance in soybean using several techniques. However, integrated use of these molecular techniques such as with CRISPR/Cas9 editing systems (base editing and prime editing) (Figure 4), TFs, genetic engineering, OMICS techniques, and QTL pyramiding and the creation and preservation of genetic diversity would be an effective step for the development of salinity tolerance in soybean. 

**Table 6:** Role of different TFs in salinity tolerance in soybean.

TFs/Genes	Role	References
*GmTGA13*	Absorption of K^+^, regulation of ions homeostasis, and activation of several stress-responsive genes	[115]
*GmLecRlk*	Increased proline content, fresh weight, and scavenging ability of antioxidants	[99]
*GmbHLH3*	Increased the accumulation rate of Cl^−^ and NO_3_^−^ in roots	[111]
*GmNAC06*	Enhanced proline and glycine betaine contents	[6]
*GmMYB46*	Mediated salt stress by the complex regulatory network	[119]
*GmMYB84*	*GmMYB84* overexpressed, and plants witnessed high germination rate, root elongation, membrane integrity, and low K^+^ level	[107]
*GmTGA17*	Enhanced chlorophyll content and proline contents under salt stress	[112]
*GmCYP81E11*	Increased the flavonoid biosynthesis	[110]
*GmDREB6*	Increased proline contents	[117]
*GmNAC15*	Enhanced activation of several genes	[113]
*GmWRKY12*	Overexpression increased proline contents under salt stress	[106]
*MsWRKY11*	Enhanced chlorophyll contents, soluble sugar, catalase activity, plant height, and pods per plant	[66]
*GmFDL19*	Higher shoot weight, plant height, and germination rate	[114]
*GmWRKYs*	Overexpressed and enhanced salt tolerance	[108]
*HD-Zip*	Improved salt tolerance by unique pattern of expression	[118]
*OsDREB2A*	Increased soluble sugars and proline contents and increased the expression of certain stress-responsive genes	[109]

## 11. Mathematical Modeling Approaches for Salinity Tolerance

There are several mathematical models used to study abiotic stress tolerance in crops. By using a modeling approach, a researcher can predict or understand the basic mechanism of salinity tolerance in crops. GWAS has been used to identify trait–marker associations and SNPs associated with a particular trait. GWAS has been performed using three basic models: the general linear model (GLM), compressed mixed linear model (CMLM), and multiple locus mixed linear model (MLMM). GLM is mainly used to reduce false association due to population structures [120]. CMLM enhances the statistical power to detect the marker–trait association as compared to other models [121]. MLMM combines the kinship matrix and Pseudo Quantitative Trait Nucleotide (QTN) to regulate the false discovery rate (FDR) [122]. GLM modifies for population structure, whereas CMLM takes both population structure and familiar affiliation into account. Both GLM and CMLM control the genomic increase efficiently and have been widely used in GWAS of soybean traits [123,124]. These are highly effective models to study salinity tolerance in soybean [80], and CMLM has especially been demonstrated as a more powerful model for association studies [121]. Random forest (RF) and artificial neural networks (ANNs) have been used to combine datasets for salinity prediction in crops [125]. Earlier, RF, support vector machine (SVM), and deep learning (DL) were applied to predict the yield-related variables in soybean, and these have been suggested as efficient models for predicting soybean crop variables. These models showed higher accuracy and prediction capabilities for soybean traits. The main purpose of these models is to validate a computational key capable of forecasting important soybean agronomic traits based on an efficient machine learning method [126]. These models have strong potential to predict the salinity tolerance in soybean based on available datasets. Another powerful model is use of ANNs, which produced more accurate and precise results for plant responses to salinity stress. ANNs are a type of nonlinear computational approach which is practical for different purposes such as clustering, forecasting, and categorizing complex systems [127]. ANNs are able to recognize the association between output and input traits and identify the inherent knowledge existing in datasets without earlier physical reflections [128]. This approach has been used to recognize the salinity tolerance indices in different wheat varieties and showed high accuracy in results [129]. Salinity tolerance is a complex mechanism, and datasets processed by these models could result in better outcomes. There is no model which can be used universally for all type of datasets. Improvement in these models would lead to better understanding of datasets and their processing. Further studies are required to explore the large-scale use of these models to study the salinity tolerance in soybean, and their accuracy and efficiency may vary depend on the type of model and dataset.

## 12. Conclusions and Future Research Directions

Salt stress reduces the soybean yield, quality, and cultivated area worldwide. The continuous decline in soybean yields is leading to an imbalance in the global food supply chain. Salt stress causes ion toxicity, reducing the soil water’s holding capacity compared to the cell water’s potential. Salt stress causes a decrease in plants’ water uptake and initiates dry conditions. Salinity tolerance is plants’ capability to resist toxic salt ions and maintain growth and yield. Salt tolerance mechanisms can be defined and grouped into many types, but not all of them can potentially work for plants’ survival. Conventional breeding methods have been used to develop soybean cultivars for salinity conditions. Complex salinity tolerance mechanism and an increased percentage of salt-affected areas hinder our way to improving plant growth and production. Breeders have chosen a powerful way to develop salt-tolerant soybean cultivars. The use of molecular techniques revolutionized the agricultural field and led to the development of several salt-tolerant soybean genotypes. QTL mapping is one of the most promising ways of improving salt tolerance by pyramiding several QTL/genes into elite cultivar; however, this technique also possesses some limitations such as an allelic assortment that segregates between parents of a particular F_2_ cross and the percentage of recombination that happens during the development of RIL population placing a boundary. This is why QTL mapping is not always a favorite choice. Genetic engineering has a crucial role in developing salinity tolerance in soybean, and transgenic soybean plants showed promising growth under salinity conditions. Despite some limitations of uses and biosafety issues, this is still considered a bold approach towards sustainable agriculture.

A loss of genetic diversity in soybean is one of the biggest hindrances in cultivating tolerant cultivars because of the loss of potential gene resources. Genetic diversity must be conserved for future breeding programs. Genetic diversity can be preserved by creating a gene bank and germplasm, which is critical for long-term use. Recent evidence revealed that soybean has lost 16 genes during domestication and increased homozygosity. The soybean population bottleneck decreases the genetic diversity because of genetic drift. Hence, CRISPR/Cas9, a novel gene-editing tool, created the hope of inducing significant genetic variation by targeted editing of the desired gene across all biological barriers. CRISPR/Cas9 use is highly recommended in the modern era of agriculture, and many crops have been improved. The development of new Cas variants and new CRISPR systems will allow for more precise editing of major genes for salt tolerance in soybean.

Transcriptome and transcription factors (TFs) analysis also successfully improved the salinity tolerance in soybean. Characterizing the soybean genome and identifying the potent TFs during salinity stress could be a possible target for CRISPR/Cas9. Recognizing new genes and their use in salt breeding programs must be ensured for developing salt-tolerant soybean cultivars. Whole-genome sequencing exposes the unexploited genetic potential in soybean, which can be fully exploited for soybean salt tolerance. These coordinated efforts can lead to a sustainable soybean yield under extreme salinity stress conditions.

## Figures and Tables

**Table 2:** Developing salt-tolerant cultivars/mutants in soybean using conventional and molecular breeding methods.

Varieties/Mutants/Transgenic Lines	Breeding Tools	References
*gmaitr36*, *gmaitr23456* mutants (Wm82 wild type background)	CRISPR/Cas9	[61]
*GmNHX5* mutants (Jidou-7 variety)	CRISPR/Cas9	[62]
*GmMYB118* mutants	CRISPR/Cas9	[63]
NIL-*J* transgenic soybean line	Genetic engineering	[23]
*PgTIP1*-transgenic lines (hybrid cultivar) 4076	Genetic engineering	[64]
cv. Liaodou 15	Genetic engineering	[25]
Transgenic lines (T_3_) with *TaNHX2* overexpression	Genetic engineering	[65]
Jackson (Ncl gene)	Genetic engineering	[59]
OX1, OX2, OX4 transgenic lines	Genetic engineering	[66]
Nannong 1138-2	Conventional breeding	[32]
Tiefeng 8	Conventional breeding	[52]
Baiqiu 1	Conventional breeding	[57]
Fengzitianandou	Conventional breeding	[57]

**Table 4:** GWAS-based identified genes for salt tolerance in soybean.

Gene	Functions	References
*GIGANTEA*	Reduced the flowering time and increased salt tolerance	[88]
*GsAKR1*	Enhanced salt tolerance during germination stage	[83]
*GmDUF4228-70*	Promoted the expression of marker genes during salt stress	[84]
*GmCHX*	Exhibited large allelic variation and improved salt tolerance	[87]
